# Asynchrony in coral community structure contributes to reef-scale community stability

**DOI:** 10.1038/s41598-023-28482-7

**Published:** 2023-02-09

**Authors:** G. Srednick, K. Davis, P. J. Edmunds

**Affiliations:** 1 School of BioSciences, University of Melbourne, Parkville, VIC Australia; 2 Department of Civil & Environmental Engineering and Earth System Science, University of California, Irvine, USA; 3 Department of Biology, California State University, 18111 Nordhoff Street, Northridge, CA 91330-8303 USA

**Keywords:** Population dynamics, Community ecology, Biodiversity, Marine biology, Physical oceanography

## Abstract

Many aspects of global ecosystem degradation are well known, but the ecological implications of variation in these effects over scales of kilometers and years have not been widely considered. On tropical coral reefs, kilometer-scale variation in environmental conditions promotes a spatial mosaic of coral communities in which spatial insurance effects could enhance community stability. To evaluate whether these effects are important on coral reefs, we explored variation over 2006–2019 in coral community structure and environmental conditions in Moorea, French Polynesia. We studied coral community structure at a single site with fringing, back reef, and fore reef habitats, and used this system to explore associations among community asynchrony, asynchrony of environmental conditions, and community stability. Coral community structure varied asynchronously among habitats, and variation among habitats in the daily range in seawater temperature suggested it could be a factor contributing to the variation in coral community structure. Wave forced seawater flow connected the habitats and facilitated larval exchange among them, but this effect differed in strength among years, and accentuated periodic connectivity among habitats at 1–7 year intervals. At this site, connected habitats harboring taxonomically similar coral assemblages and exhibiting asynchronous population dynamics can provide insurance against extirpation, and may promote community stability. If these effects apply at larger spatial scale, then among-habitat community asynchrony is likely to play an important role in determining reef-wide coral community resilience.

## Introduction

In every major biome, decadal-scale variation in community structure reveals the imprint of anthropogenic disturbances^1,2^, much of which is caused by climate change^3,4^. These effects are diverse in provenance and consequences, but through changing population sizes leading to extirpations, range expansions, and invasions, many communities exhibit signs of reduced resilience and stability^5,6^. Although the ecological impacts of disturbances are extensive^7^, on a local scale (i.e., ≤ 20 km)^8^ the responses can be heterogeneous^9,10^. Local-scale variation can seem trivial relative to the global biological responses to the Anthropocene epoch, but variation at small spatial scales can provide insights into the ecological changes taking place at the largest spatial scales^11,12^, especially when the organisms of interest exhibit variability in conditions across multiple spatial scales. For example, in observational studies^13,14^ spatial heterogeneity in the response of populations or communities to conditions across relatively small (400 m^2^ to 3 km) scales can be transmitted at larger spatial scales (> 1.3 to ~ 64 km).

Where regional-scale degradation of communities suggests impending ecosystem collapse (e.g., coral reefs^15^), small scale heterogeneity in these effects can ensure that patches of the community differ in the extent to which they are degraded^16,17^. Assemblages of taxa with interdependent demographic rates within a location, hereafter “communities”^18,19^, can be separated by spatial barriers (e.g., reduced connectivity by physical barriers) that reduce interactions and promote heterogeneity in environmental conditions, yet allow propagule exchange. Spatial networks of community patches can function as a metacommunity^19^ in which population dynamics are associated among patches, but potentially can fluctuate out of phase^9^. In these networks, degraded patches can benefit from organisms dispersing to them from less-degraded patches at local (within habitat) and regional scale (across-habitat)^20,21^, thereby promoting metacommunity persistence^17^. These relationships have been formalized through the concept of asynchrony among communities in species abundance^22^. Within the spatial insurance hypothesis^23^ asynchrony arising from population sizes that fluctuate asynchronously among localities connected by larval dispersal can stabilize temporal variation in community structure (e.g., when species or localities compensate for declines in abundance)^23,24^. Spatial insurance has been relatively well studied in the marine environment^25–29^ where larval connectivity^30,31^ and the flow of seawater^32^, facilitate positive synergy in the dynamics of populations in different locations, and promotes community stability^27^.

Despite advances that have come from interpreting community dynamics through the spatial insurance hypothesis^23^, wider adoption of the concept has been impeded by the slow emergence of a consensus regarding the best means to achieve this outcome^33^. It has been proposed that the spatial insurance hypothesis can be evaluated by quantifying spatiotemporal variation in population dynamics for multiple species (i.e., synchrony), the temporal variation in their community structure, and genetic connectivity among spatially separated populations^23,34,35^. The extent to which populations of multiple species respond in similar ways to the same environmental conditions defines community synchrony^34,36^, with spatial variability in environmental conditions (e.g., temperature) promoting asynchronous dynamics. Stability, the degree to which community structure changes over time, can be evaluated through “community variability” (CV)^36,37^, which can be manifested as spatial insurance through the presence of larval connectivity (e.g., mediated by wave-driven transport) among spatially asynchronous community dynamics across habitats. While these concepts have been successfully applied to terrestrial communities^38^, and some marine species^29,39^, they have not been applied to coral reefs that have become the poster child for ecosystem collapse. Here we apply the framework of the spatial insurance hypothesis^23^ to a coral reef to explore the implications of among-habitat asynchrony in community dynamics with respect to the capacity to stabilize among-habitat variation in coral communities.

Since the 1970s, the coral reefs of Moorea have experienced multiple cycles of disturbance and recovery of benthic communities^40–44^. Major disturbances have been created by outbreaks of the corallivorous seastar *Acanthaster solaris* (hereafter COTs)^43^, cyclones^40^, and coral bleaching^45,46^, which have caused rapid and large declines in coral cover^41^. These have been followed by the return of coral cover to (or in excess of) pre-disturbance values within 8–10 years^41^. While these trends have changed the relative abundance of coral taxa indicating reduced recovery to the pre-disturbed state ^40,41^, coral cover on the fore reef of Moorea has been resilient to disturbances^47–49^, notably in comparison to other coral reefs throughout the Indo-Pacific^50^ and Caribbean^51^. Following the 2003–2010 outbreak of COTs^43^, and a cyclone in 2010, the fore reef (10-m depth) on the north shore regained coral cover at an unprecedentedly high rate^52^. The upward trajectory of coral cover was interrupted by bleaching in 2019^53^, but by August 2020 the mean coral cover at 17-m depth on the north shore had reached 31% (P.J. Edmunds, unpublished data), which is higher than the mean coral cover for the Indo-Pacific in 2003 (22%)^50^. While recovery of coral cover in fore reef habitats in Moorea following disturbance has been high, these patterns are spatiotemporal heterogeneous across habitat types, with fore reef habitats, in general, exhibiting faster recoveries than lagoon habitats^54^. Understanding the drivers of high coral community resilience in the few locations where it has been recorded in recent years^55^ has become a major focus of coral reef science.

Our analyses of coral community structure in Moorea addresses spatiotemporal variation among four habitats (i.e., fringing reef, back reef, fore reef 10-m depth, and fore reef 17-m depth) separated by < 1 km along a transect parallel to the direction of net seawater flow across the reef^56^. Using annual surveys from 2006 to 2019, we tested two hypotheses and used the outcome to explore the role of environmental conditions in driving asynchronous changes in coral communities. First, we hypothesize that different habitats will exhibit spatiotemporal variability (i.e., asynchrony) in coral community structure, as a result of variation in environmental conditions and variable disturbance regimes^40,41,54^. Secondly, and assuming the first hypothesis would be accepted^57,58^, we hypothesize a positive relationship between metacommunity synchrony (phi) and variability (CV) among habitats, indicating that temporal synchrony in community structure across habitats has a destabilizing effect on metacommunity stability. To evaluate possible causation of spatial insurance in this system, we explored the associations between community synchrony and CV, and physical environmental conditions (seawater temperature and cross-reef, wave-driven seawater transport). We focused on temperature because of its role in mediating coral performance^59^ and bleaching^60^, and wave-driven seawater transport because of its capacity to mediate coral community structure through larval transport^61^. Finally, we discuss this framework and its application at a single site as a proof-of-concept for use in assessing the contribution of spatiotemporal heterogeneity in community structure in promoting spatial insurance effects in the coral communities of tropical reefs.

## Materials and methods

### Overview

Our analyses are based on coral communities (scleractinians and the hydrocoral, *Millepora*) resolved to genus (and one family) in a conservative interpretation of the taxonomic resolution supported with photoquadrats^62^. Photoquadrats were recorded in four habitats at a single site on the north shore of Moorea, where they are physically connected by wave-driven, cross-reef seawater flow^56^. This system of habitats at a single site exploited existing environmental monitoring^63^ and provided a tractable system in which to test our hypotheses, although it sampled habitats differing in coral generic richness. The differences in richness potentially indicates that the fringing reef has a reduced capacity to supply coral propagules from diverse genera to other habitats, since it was dominated by massive *Porites* and *P. rus* (accounting for > 98% of the coral cover over 2006–2019).

### Ecological sampling

**Figure 1:**
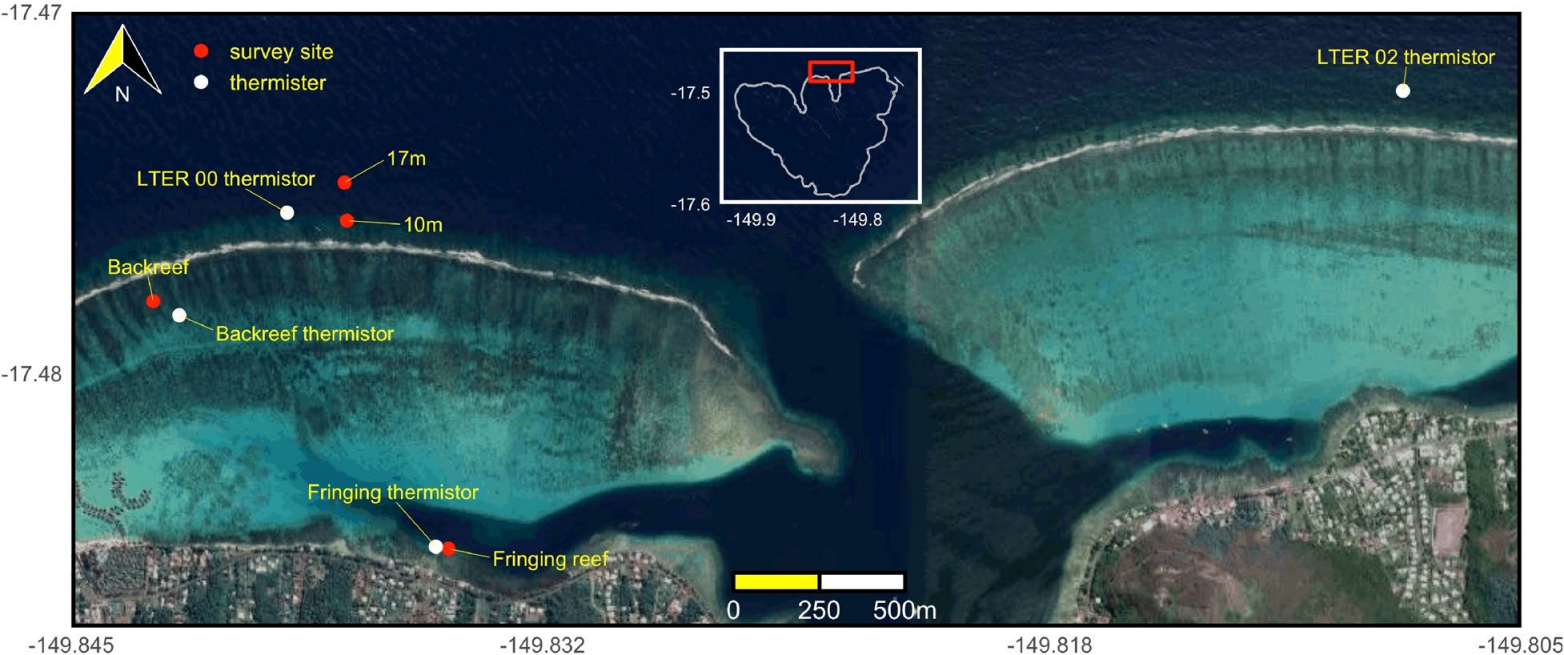
Map showing the four habitats (fringing reef, backreef, 10 m, and 17 m on the fore reef) and thermistor locations in Moorea, French Polynesia. Photo credit: Google Earth.

Coral community structure was analyzed in four habitats, the fringing reef, back reef, fore reef 10-m depth, and fore reef 17-m depth, that were sampled at the LTER1 site on the north shore of Moorea (Fig. 1). The four habitats were sampled midway along the 16-km long north shore, and were positioned across a ~ 1 km wide portion of the shore, beginning at the fringing reef and ending at the fore reef. At this site, the reef crest is ~ 900 m from the land, the back reef occupies a shallow lagoon that is mostly < 4-m depth, and the fore reef extends to > 100-m depth within ~ 100 m of the reef crest.

This study began in 2006, one year after the permanently marked sites were established, and extended annually with sampling in April/May to 2019. The fringing reef (2–5 m depth) and fore reef (10-m and 17-m depth) are each sampled using a 50 m transect along the isobath. Along this transect, photoquadrats (0.5 × 0.5 m, n = 40) were randomly located in 2006, and the same positions are sampled annually. The back reef is spatially heterogeneous and, therefore, sampling focused on five bommies that were haphazardly selected and permanently marked in 2006. The five bommies are ~ 130 m from the reef crest, and each is sampled using quadrats (0.5 × 0.5 m) placed randomly along four cardinal axes 5 m in length. With 5 quadrats along each axis, each bommie is sampled with 20 quadrats year^−1^.

Photoquadrats were recorded using digital cameras attached to strobes (Nikon SB105) and held perpendicular to, and at a fixed height above, the reef. Cameras were replaced throughout the study to increase resolution from 6.1 MP (Nikon D70), to 16.1 MP (Nikon D7000), and then 36.3 MP (Nikon D810), and allowed benthic objects > 5–10 mm diameter to be identified. For sampling on the fringing reef and fore reef, the cameras were fitted with a zoom lens (Nikkor AF S, f3.5-G ED 18–70 mm DX, or f3.5-4.5G ED 18–35 mm FX), but a wide-angle lens (Nikkor AF 16 mm FX) was used in the back reef where the shallow depth required the camera-subject distance to be shorter. Images were analyzed using CoralNet software^64^ with manual annotation using 200 randomly-located dots image^-1^, under each of which the substratum was resolved to coral genus/family and scored as percentage cover. Corals were identified based on gross morphology (after Veron^65^). The present study focuses on a taxonomically diverse group of organisms that cannot be resolved to species in photographs and, for the most part, taxonomic resolution mostly was to genus (and one family). However, given the taxonomic and functional diversity among these taxa (14 taxa with different morphologies and life-histories), together with the clear spatiotemporal variation in community structure across habitat types, suggests that the taxonomic resolution employed here are adequate in describing patterns of spatiotemporal variation in coral community structure.

### Physical environmental conditions

Seawater temperature was recorded using logging thermisters (Seabird Electronics SBE39, ± 0.002 °C) mounted to the benthos to sample every 20 minutes^66^. Loggers periodically were replaced over the 14 year study, and the records were used to assemble a near-continuous time series for each habitat. The thermisters were installed ≤ 100 m from the locations at which benthic community structure was measured in each habitat, although occasional equipment malfunction required data gaps to be filled with data from other thermisters mounted at the same depth and in the same habitat but further away (i.e., ~ 3.5 km) on the north shore (described in results). The forereef temperature sensors closest to the 10 m and 17 m benthic community survey sites (LTER00, Fig. 1) were not installed until 2010, therefore forereef temperature sensors on the north shore (LTER02 see Fig. 1) were used to characterize the forereef temperature. Seawater temperature was compared between the LTER00 and LTER02 sensors at the same depth for four years when they were operating concurrently (April 2010- April 2014). The difference in mean temperature between the LTER00 and LTER02 over this period is < 0.03 °C, while the difference in standard deviation is < 0.02 °C between sites. Seawater temperature at each habitat site was used to estimate daily average mean seawater temperature, maximum (*T*_*max*_) and minimum (*T*_*min*_) daily temperature, and the diurnal temperature range (DTR = *T*_*max*_–*T*_*min*_) over the 13 year time series^67^.

Waves were measured at the LTER01 fore reef site using a bottom mounted wave-tide meter (Seabird Electronics, SBE-26), which measures significant wave height (*H*_*sig*_) and dominant wave period (*T*_*w*_). Wave data are available for the entire 13 year time period with the exception of a year-long gap in 2011 (wave statistics not estimated for this year), and shorter gaps in 2010 and 2018. Wave energy flux is an estimate of the mean transport of wave energy through a vertical plane parallel to the reef crest and can be calculated as,

$$P = \rho g^{2} H_{sig}^{2} T / 64 \pi$$

where *P* is energy flux (kW m^−1^), *ρ* is the mean seawater density (~ 1028 kg m^−3^), and *g* is gravitational acceleration (9.8 m s^−2^)^68^. Wave energy flux was summed from September to December in each year, corresponding to the spawning period for *Acropora* spp. in Moorea^69^.

### Statistical analyses

The coral community structure in each habitat was described with genus/family resolution and visualized as mean coral cover (± SE, n = ~ 40 quadrats/y in the fringe and fore reef, n = 5 bommies in the back reef) using scatter plots. The effects of time on coral cover were not tested with inferential statistics as they have been described elsewhere^52,54,70^, and because the trends are revealed by the differences in mean cover and SE. Multivariate coral community structure over time in each habitat was evaluated using 2-dimensional ordination prepared with non-metric multidimensional scaling (NMDS) employing 20 restarts or until stress stabilized at < 0.02^71^. Percentage coral cover by genus/family was prepared as a resemblance matrix using Bray Curtis dissimilarities before visualizing using NMDS. Homogeneity among habitats in multivariate variation over time (Hypothesis 1) was tested using two factor PERMANOVA in which habitat and time were fixed effects and the dependent variables consisted of measurements of coral cover by taxon. The test of Hypothesis 1 focused on the significance of the interaction between habitat and time. The analyses used 999 permutations and were based on Bray–Curtis similarities with a dummy variable with value of 1 added to each sample to account for zero-inflation (sensu Clarke et al.^72^).

To test Hypothesis 2, we quantified variation among taxa between years and among habitats with community synchrony and CV^34^, and examined their covariation. These metrics were calculated annually to allow for greater resolution in identifying periods of variation. Community synchrony scales from 0 to 1, with unity showing that all taxa changed in abundance in identical ways, and zero indicating that taxa varied in abundance in dissimilar ways, and is calculated as

$$\varphi = \frac{{\sigma (x_{T} )^{2} }}{{\left( {\mathop \sum \nolimits_{i} \sigma_{xi} } \right)^{2} }}$$

where $\sigma_{x}$ is variance in cover of each taxon across habitat types^36^. Heterogeneous variation in abundance among taxa raises the possibility that a decrease in the abundance of one taxon is compensated by the increase in abundance of another taxon^31^. CV scales from 0 to > 1, with high values indicating large differences in community structure between times (i.e., low stability), and is calculated as:

$$CV = \frac{\sqrt \sigma}{u}$$

where $u$ is mean total cover of all taxa across habitat types^22^. Community synchrony and CV were calculated from coral cover by genus/family across habitats with annual resolution (described in Supplementary Materials). Variation over time in community synchrony and CV was visualized with scatterplots, and major axis regression (MA) model II linear regression was used to test the association between community synchrony and CV as an indicator of the presence of trends consistent with spatial insurance (sensu Yachi and Loreau^24^). MA regression was used because CV and community synchrony are random effects and are dimensionless^71^. The inclusion of community synchrony in the calculation of CV^23,24^ leads to an intuitive association between the two. We follow the framework of Loreau et al.^23^ and subsequently Wilcox et al.^35^ in our interpretation of evidence of association between the two metrics in concert with physical environmental attributes in understanding drivers of the association.

Finally, we explored the association between the emergent properties of coral community structure (i.e., community synchrony and CV) and two aspects of the physical environment (i.e., temperature and wave energy flux). To broaden the capacity to evaluate concordant synchrony of variation in the two domains, we computed both mean temperature and diurnal temperature range (DTR) at each habitat and evaluated how spatial variation in temperature contributed to spatial synchrony in coral community dynamics. Additionally, we explore and discuss how mean wave energy flux could promote spatial insurance to the extirpation of taxa by promoting connectivity among habitats.

Analyses on coral community structure and figures were generated using R-studio^73^ (version 2021.09) with the packages ‘Vegan’^74^ for multivariate analyses and ‘tidyverse’^75^ for graphics and data curation. Physical data were analyzed using Matlab (version R2021a). The code for these analyses is archived at https://github.com/gsrednick/moorea_asynchrony_stability.

## Results

### Overview

**Figure 2:**
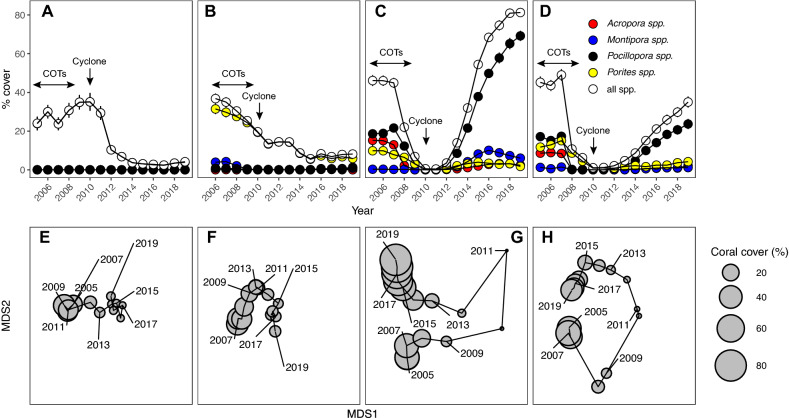
Changes over time in coral cover and community structure across habitats at LTER 1: (**A**,**E**) fringing reef, (**B**,**F**) back reef, (**C**,**G**) 10-m depth on the fore reef, and (**D**,**H**) 17-m depth on the fore reef. (**A**–**D**) Mean cover (± SE) of the most abundant corals, and (**E**–**H**) non-metric multidimensional scaling (NMDS) of coral community structure over time, with circle size showing the summed percent cover for all corals. For fringe and fore reef habitats, N = ~ 40 year^−1^, and for back reef, N = 5 year^−1^.

In 2006, the four habitats each had a mean coral cover of 30–46%, and their coral assemblages differed in relative and absolute abundances of coral taxa (Fig. 2A–D). Mean (± SE) coral cover in the fringing reef was 30.0 ± 3.5% (Fig. 2A). In the back reef, mean coral cover in 2006 was 36.8 ± 4.0% (Fig. 2B), and was composed of 8 taxa, of which the five most common were *Porites*, *Montipora*, *Pocillopora, Pavona*, and *Millepora*, which together accounted for 99.5% of the coral cover. On the fore reef, mean coral cover at 10-m and 17-m depth in 2006 was 46.0 ± 2.7% and 43.6 ± 2.9% (Fig. 2C,D), and was composed of 12 and 13 taxa, respectively. *Acropora*, *Pocillopora,* and *Porites* accounted for 95% of the cover at 10-m depth, and 84% of the cover at 17-m depth. Coral cover in all four habitats changed over time between 2006 and 2019, with large declines in the fringing and back reef habitats, and large declines followed by rapid recovery at 10-m and 17-m depth on the fore reef.

In the fringing reef, mean coral cover increased from 2006 (30.0%) to 2010 (35.1 ± 4.6%) before declining to 3.7 ± 0.9% by 2014 and 2.4 ± 0.7% by 2017; by 2019, it had increased to 4.2 ± 1.0%. From 2006 to 2019, massive *Porites* accounted for > 82% of the coral cover, although small amounts of *Acanthastrea* (e.g., 2.4% relative to overall coral cover in 2017), fungids (5.5% in 2019), and *Montipora* (4.8% in 2016) were found in several years.

In the back reef, mean coral cover declined almost linearly from 2006 (36.8%) to reach 8.1 ± 1.3% in 2019, with most of the decline caused by the death of *Porites*. In 2006, *Porites* accounted for 86% of the coral cover, but by 2019, this declined to 72%. Fifteen other taxa were encountered in the back reef, and of these, the most common were *Montipora* and *Pocillopora*. In 2006, these two genera accounted for 10.7% and 2.2% of the coral cover, respectively, but by 2019 their coverage had changed to 10.1% and 16.1%, respectively.

Coral community dynamics were more complex on the fore reef compared to the back and fringing reefs. At 10-m and 17-m depth, coral cover in 2006 (46.0% and 43.6%, respectively) began rapid declines within three years as COTs consumed coral, and by 2009 mean coral cover had declined to 6.9 ± 1.5% and 6.2 ± 1.2%, respectively. The following year, Cyclone Oli (February 2010) removed most dead-in-place coral colonies, and reduced coral cover to 0.5 ± 0.4% and 1.0 ± 0.3% by April 2010, at 10-m and 17-m depth, respectively. Thereafter, coral cover in the 10-m and 17-m depth habitats increased to 81.3 ± 5.6% and 35.0 ± 5.9% by 2019, respectively. At 10-m depth and prior to 2010, coral cover included 14 taxa, of which *Pocillopora*, *Acropora* and *Porites* accounted for an average of 48%, 18%, and 28%, respectively. Following 2010, taxonomic richness increased over 2011 to 2019 (12 taxa and fungids), and the relative abundance of taxa differed from that recorded before 2010. *Pocillopora* accounted for > 25% of the coral cover over this period, with *Pocillopora*, *Acropora* and *Porites*, accounting for an average of 66%, 3% and 9% of the overall coral cover, respectively. At 17-m depth, coral cover before 2010 was composed of 15 taxa, of which *Pocillopora*, *Acropora* and *Porites*, accounted for a mean of 18%, 10% and 53%, respectively. Richness remained the same over 2011–2019 (14 genera and fungids), but their relative abundance varied. *Pocillopora* accounted for > 5% of the coral cover throughout this period, with *Pocillopora*, *Acropora* and *Porites* accounting for an average of 52%, 3% and 26%, respectively.

The abundance of coral taxa described temporal variation in multivariate assemblages that were unique to each habitat (Fig. 2E–H). In the fringing reef and back reef, multivariate community structure showed incremental changes among years with 2019 differing from 2005 with respect to community similarity (Fig. 2E–H). In the back reef, although coral cover consistently declined over time (Fig. 2B), multivariate community structure in 2006 and 2019 was more similar than in 2006 versus 2013, or 2006 versus 2015. On the fore reef at 10-m and 17-m depth (Fig. 2G,H), variation in multivariate community structure was striking as revealed by the wide separation of community states in 2-dimensional ordination in 2006 versus 2010 and 2011. These changes corresponded to the large loss of live coral associated with COTs and Cyclone Oli (Fig. 2C,D). Over the 14 year study, the multivariate community structure at 10-m and 17-m depth trended to a complete recovery of coral composition as the community states converged and the similarity between 2006 and 2019 rose to 0.61 and 0.37, for 10 m and 17 m, respectively. From 2014 to 2019, multivariate community structure at 10-m depth started to diverge from the community structure recorded in 2006, as revealed by the increasing separation between community states between 2006 and 2019. The divergence in community state among these years was associated with the rising abundance of *Pocillopora,* which exceeded pre-disturbance cover after 2014 (10-m depth) or 2017 (17-m depth) (Fig. 2D,E).

### Hypothesis 1. Asynchrony among habitats

**Figure 3:**
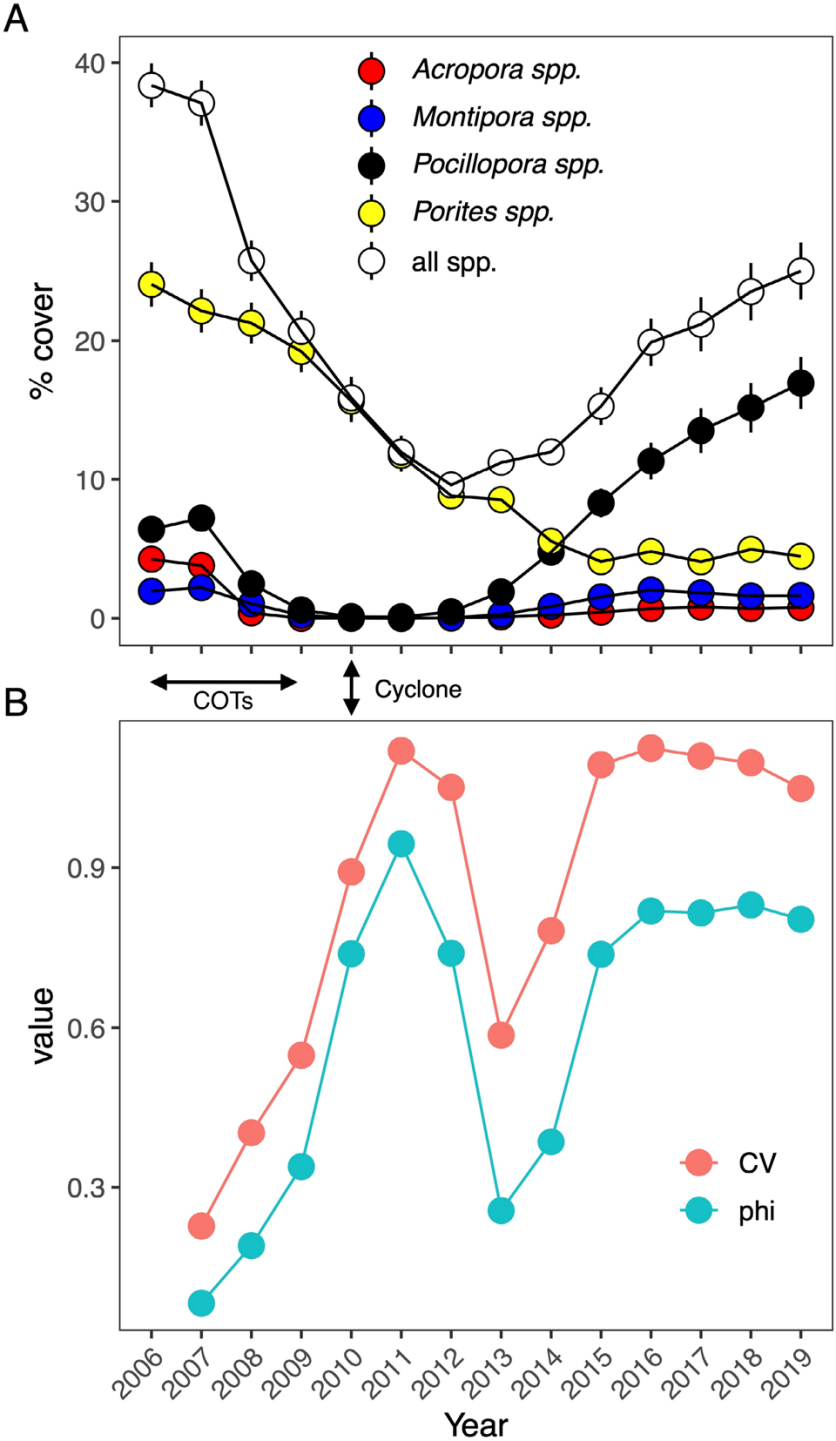
Coral cover from 2006 to 2019 at LTER1, pooled among habitats: (**A**) mean (± SE, n = 4 habitats year^−1^, SE bars smaller than symbol size where they are not shown) percent cover of the four most common corals that account for 43–95% of the coral cover in each habitat, and (**B**) synchrony (phi) and community variability (CV) over time.

Of the 19 taxa that were detected in the four habitats, the most common were *Acropora*, *Montipora*, *Pocillopora*, and *Porites*. When averaged over time and habitats, these taxa accounted for 95% of the coral cover, although their cover varied over time in ways that were unique to each habitat (Fig. 2A–D). Reef-wide (i.e., pooled among habitats) coral community dynamics captured the aforementioned trends in habitat-specific coral community dynamics. An overall trend for a decline and then increase of coral cover was an emergent property of the growth of *Pocillopora*, persistent declines in cover of *Porites* and *Acropora*, and a slight upward trend in cover of *Montipora* (Fig. 3A). The heterogeneous trends in coral cover over time and among habitats are reflected in the strong interaction between Year and Habitat in the statistical analysis of multivariate coral community structure (Pseudo-F = 118.16, df = 3, 3106, p_perm_ = 0.001).

The heterogeneous variation over time in coral cover by taxon among habitats is measured by community synchrony, which varied among years from 2006 to 2019 (Fig. 3B). Variation in percentage coral cover over 2006–2007 was almost fully asynchronous among habitats (i.e., community synchrony approached zero), showing that the coral taxa were changing over time in different ways in each habitat. Asynchrony transitioned to near complete synchrony (i.e., community synchrony approached 1) over 2007–2011 (Fig. 3B) as the coral community dynamics were dominated by large declines in coral cover in all four habitats (Fig. 2A–D). From 2015 to 2016, synchrony was high (community synchrony = 0.82) as coral community dynamics on the fore reef were influenced by large increases in coral cover, and dynamics in the back reef and fringing reef were relatively stable (Fig. 2A–D). Overall, synchrony among habitats in the coral communities tended to decline immediately post-disturbance (2011 to 2012), indicative of variability in community structure among habitats, and then increased in 2013 as coral cover increased at outer reef habitats (Fig. 3B).

The extent to which the taxon-specific coral cover was stable over time among habitats, was captured by community variability (i.e., CV), which changed over time (Fig. 3B). Over 2006–2007, coral cover was stable among habitats when CV approached zero. CV increased over 2007–2008 as the coral taxa responded in similar ways to prevailing conditions (i.e., community synchrony ~ 1), and trended downward, post disturbance, indicative of variation in community structure among habitats. CV then increased due to year-to-year variability among coral taxa across habitats because of increasing *Pocillopora* cover from 2013 to 2015 at 10-m and 17-m (e.g., CV = 0.55 over 2007–2008 but CV = 1.05 over 2018–2019), after which CV plateaued and began decreasing in 2016 that extended to 2019.

### Hypothesis 2. Association between community synchrony and CV

**Figure 4:**
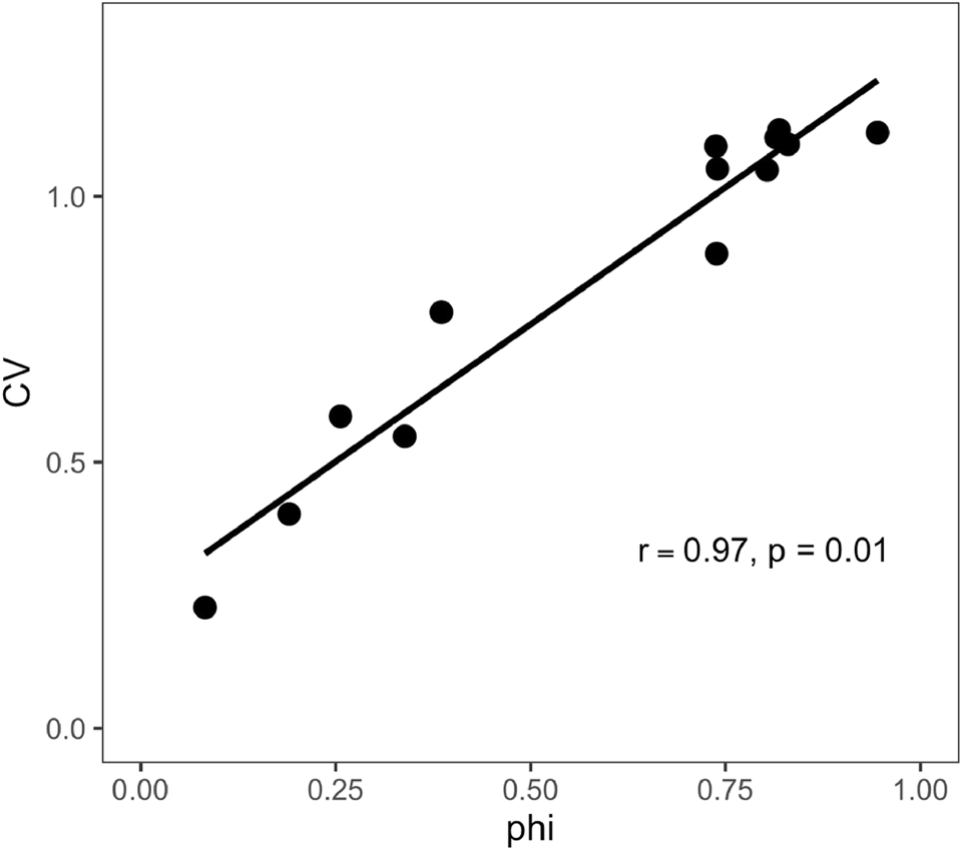
The relationship between community variability (CV) and synchrony (phi) for the reef-wide coral community over 13 years from 2006 to 2019. Line is fitted by model II major axis regression of CV on phi.

The divergence in trajectories of changing coral cover by taxon that occurred in the fore reef versus the back reef and fringing reef (Fig. 1) was associated with rising asynchrony (declining community synchrony) and increasing stability (declining CV) of coral cover among habitats (Fig. 3B). Community synchrony and CV were significantly and positively associated (r = 0.97, df = 11, *p* = 0.01; Fig. 4), with Model II regression showing that variation in community synchrony accounted for 94% of the variation in CV.

### Association between coral community structure and the physical environment

**Figure 5:**
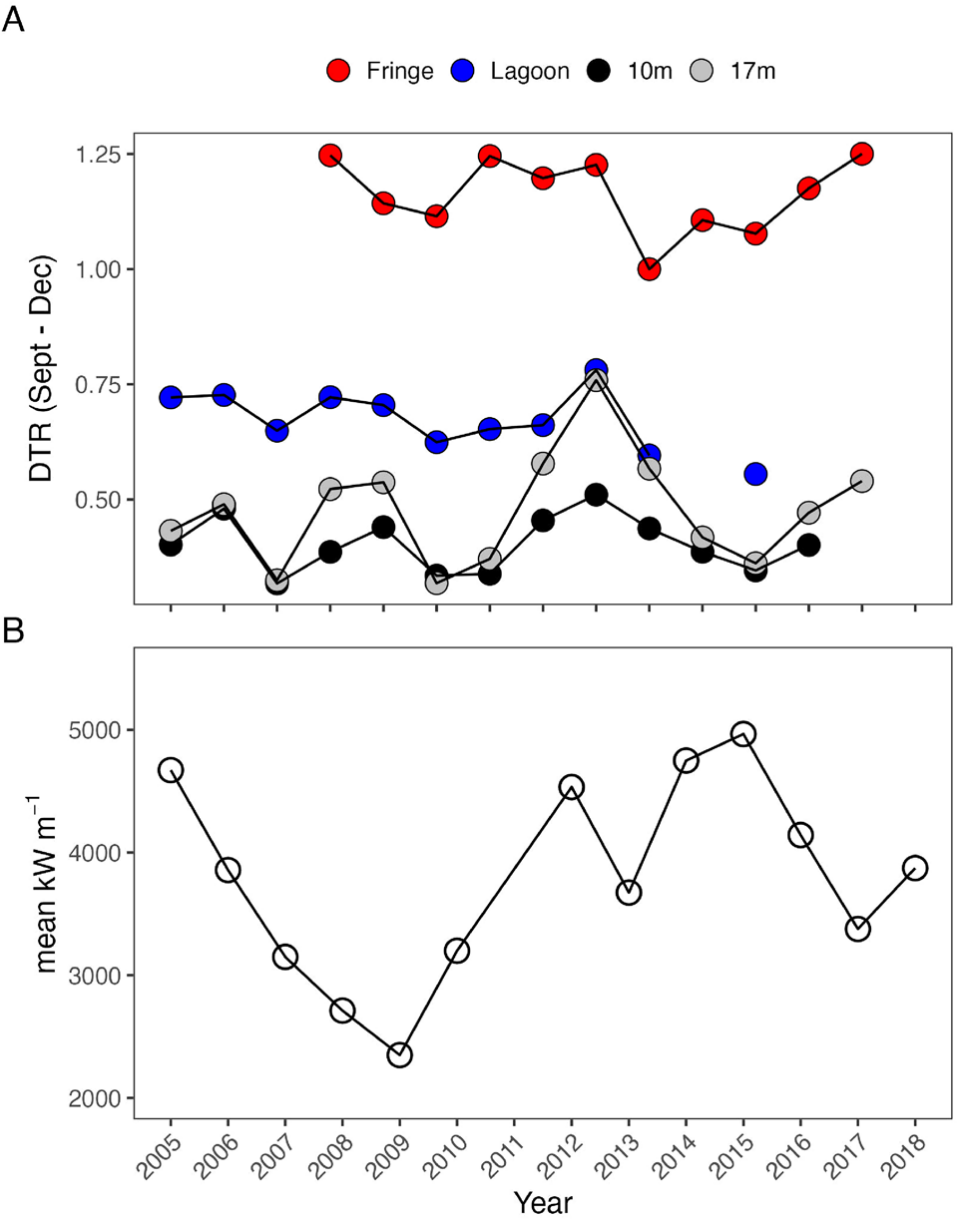
Variation in (**A**) diurnal temperature range °C (DTR) during the inferred period of coral spawning and pelagic larval duration (September to January) across habitats, and (**B**) mean wave energy flux (kW m^−1^) across the reef crest on the north shore during the same period. Missing points indicate periods for which DTR or wave energy flux were not calculated due to gaps in the dataset.

Annual average bottom temperatures exhibited a high degree of synchrony (phi > 0.97) between habitats, and mean bottom temperatures in each habitat over the 14 year record were very similar, ranging from 27.73 °C at the 17 m fore reef, to 27.86 °C in the fringing reef. The diurnal temperature range (DTR) varied among habitats (Fig. 5A). Mean DTR over the full record in the fringing reef (1.07 °C) was more than three times larger than at the 10 m fore reef habitat (0.33 °C), and synchrony of DTR between habitats was lower (0.41 < phi < 0.57) than for annual mean temperature.

Wave energy flux averaged over the inferred period of coral spawning and pelagic larval duration (PLD; September–January) (Fig. 5B) exhibited high temporal variability over the study, with peaks in 2005, 2012, 2014, and 2015 (i.e., every 1–7 years). The period of declining coral cover from 2007 to 2012 was associated with a decrease in wave energy flux averaged over the coral spawning and PLD period combined (50% decrease, Fig. 5B), while the period of rising coral cover from 2012 to 2018 was associated with a general increase in wave energy flux, peaking at 4,966 kW m^−1^ in 2015.

## Discussion

### Overview

Understanding the factors determining community stability and resilience is an important goal of ecology, particularly to assess how communities respond to the disturbances characterizing the Anthropocene Epoch^76–80^. Spatial insurance^23,24^, which has emerged as a promising hypothesis of use in these efforts^81,82^, posits that diversification among assets (e.g., species or individuals) stabilizes performance and decreases the likelihood of local extirpation (i.e., in ecology, community resilience)^83^. In this hypothesis, multiple proximal mechanisms (e.g., spatio-temporal variability in environmental conditions) contribute to (and characterize) the responses of a diverse biological portfolio to environmental conditions^84^. Tropical coral reefs provide an interesting system in which to test for these effects, because they represent a complex ecosystem of near-unrivalled diversity ^85,86^, their depth-dependent geomorphology creates strong environmental gradients across which this diversity is expressed^87^, and they face substantial threats from climate change^13,43^.

The coral reefs of Moorea dramatically changed from 2006 to 2019^52,70,88^, with COTs and Cyclone Oli^43^ serving as conspicuous disturbances that caused large changes in coral community structure on the fore reef. Here, coral cover declined from 45% in 2006, to near-zero in 2010 and 2011, before increasing to ~ 58% by 2019 (Fig. 2). The rapid recovery of the coral community in this location has been attributed to a variety of effects, including high and density dependent recruitment of *Pocillopora* spp.^52,70^, increased fish herbivory that prevented macroalgal blooms^45^, and ecological rescue and response diversity affecting pocilloporid corals^52^. While recovery of coral cover in fore reef habitats in Moorea following disturbance has been rapid and near-complete (in some places, coral cover on the fore reef in 2019 was higher than that before the COTs outbreak), these patterns are spatially and temporally heterogeneous across habitat types^88^. At the present study site where the back reef and the fringing reef have been repeatedly sampled at permanently marked locations, coral cover has declined without recovery^88^. The causes of declines in coral cover in these habitats at our study site are not clearly related to either the COTs outbreak of 2003–2010, or Cyclone Oli, both of which strongly affected the fore reef^41,43^. Moreover, the changes affecting coral cover in the back reef and fringing reef over 2006–2019 have occurred asynchronously (i.e., at different times and at varying rates compared to the fore reef). By highlighting asynchrony in coral community dynamics among four adjacent habitats on the north shore of Moorea, our results suggest that the coral community might benefit from among-habitat insurance effects^23^. If this interpretation is correct, it is possible that reef-wide asynchrony among local coral communities could modulate ecological stability of the coral community at kilometer-scale.

Based on the present analyses, it is not our intent to suggest that the results from a single site apply to the reef system surrounding the ~ 50 km shore of Moorea, nor do we intend to imply cause-and-effect in the association between synchrony and stability. Moreover, we do not argue that asynchrony in coral cover is an indicator for functional resilience in coral communities, which is yet to be explored (*but see* McWilliam et al.^44^ for functional diversity and redundancy). Our study provides, however, a proof of concept analysis exploring the utility of spatial insurance as a mechanism promoting reef-wide ecological stability of coral communities. Our analyses make a compelling case that spatial insurance could play a role in mediating coral community resilience in Moorea. We posit that the causal origin of this association lies in the spatiotemporal heterogeneity of physical environmental conditions and the ways in which they could modulate coral demography, for example, through recruitment and coral mortality^89^.

### Heterogeneity in coral community dynamics (Hypothesis 1)

Our results reveal the high degree of spatiotemporal heterogeneity in coral community structure in four habitats separated by < 1 km. Such variation has been reported from coral reefs for decades^57,88,90^, especially with respect to adjacent habitats^91^, and the magnitude of spatial variation in coral cover reported here (26% difference in coral cover between adjacent habitats in 2009) is similar to the magnitude of spatial variation reported from other locations^92^. Spatial heterogeneity in community state, and differences in the rates at which it can change in adjacent locations, can arise from multiple mechanisms. However, a long-standing explanation has been that the successional clock^93^ is reset by disturbances at different times in adjacent locations and/or habitats^94^. This could be due to environmental disturbances that are spatially heterogeneous on a scale commensurate with the spatial separation among habitats. For example, intense tropical storms that can decimate shallow reefs in exposed locations, while leaving adjacent areas in deeper water or sheltered locations virtually intact^95,96^. By creating a spatial mosaic of damage, recovery from damage, and differential timing on the successional clock, community dynamics inherently can remain asynchronous among closely-spaced locations.

To date, the ecological significance of spatial variation in the timing of declines in coral abundance, the commencement of increases in coral cover following disturbances, or taxonomic variation in these rates, have not been considered as mechanisms contributing to coral community stability. Examination of these effects in other systems indicates that spatial heterogeneity in population and community dynamics can influence population or community stability. For example, spatially asynchronous dynamics among herring populations along a 300 km archipelago^29^, fishes in large-scale Atlantic trawl surveys^97^, and plant metacommunities^35^ ranging in size 0.03–10 m^2^, all promote aggregate stability in abundance or biomass. While the demonstration that coral communities in adjacent habitats differ in instantaneous state (i.e., coral cover by genus), as well as the rate and timing of changes of cover, supports well known spatial trends in coral community structure, it is still relatively new for this field^98–100^ to suggest that coral community asynchrony can play a role in modulating metacommunity stability.

### Association between synchrony and stability (Hypothesis 2)

The positive association between community stability (CV) and synchrony for the present coral communities supports our second hypothesis, and suggests that asynchronous dynamics could contribute to reef-wide stability of the coral community^101^. This possibility is consistent with results from other ecosystems, in which community synchrony and CV are strongly and positively associated, for example, for grassland plants in the desert of New Mexico^102^, and among North American breeding-bird species^103^. Similar relationships have also been reported from a floodplain habitat near Thuringia, Germany^84,104^, freshwater plankton in Michigan^105^, and marine macroalgae in central California^27^. The finding that synchrony and CV are related is not surprising given their similar mathematical basis, and abundant evidence of a positive association between the two. However, the present study describes evidence of asynchrony across habitat types, and we explore potential drivers and consequences of asynchronous patterns in coral community structure. While it was beyond the scope of the present study to test for causality in the association between community synchrony and CV, there are at least two non-exclusive mechanisms that could lead to the asynchrony among habitats that we have revealed.

First, spatial variation in environmental conditions could favor decoupled population and community dynamics among locations distributed over a spatial scale commensurate with the spatial scale of variation in environmental conditions^24,84,106^. Demographic stochasticity for corals on the reefs of Moorea could promote asynchronous variation in coral abundance among habitats, for example, through changes in abundance driven by stochastic delivery of coral propagules within, and among, habitats^107,108^. Synchrony might be favored among sites sharing common environmental conditions and having similar abundances of coral larvae, while asynchrony would be favored among sites exposed to dissimilar conditions and having heterogeneous larval supply. Under this mechanism, the spatial scale of biological and environmental heterogeneity would be expected to vary depending on the environmental conditions causing the communities to change. For example, severe storms are likely to create heterogeneous effects over a scale of kilometers^95,109^, whereas the effects of marine heatwaves are likely to be consistently expressed over 10–100 s of kilometers^89^. Second, spatial variation in the strength of biotic interactions among coral taxa, for example, habitat-specific variation in the intensity of spatial competition among corals, could decrease synchrony among habitats in variation of abundance among taxa^37^. For example, low densities of coral colonies in one (or more) habitat(s) would attenuate the effects of spatial competition among corals (because they are less likely to contact one another), thus avoiding depressed colony growth and the need for vertical extension to circumvent spatial competition among crowded colonies^110^. In habitat(s) where coral colonies occur at high densities and contact one another, competition for space is likely to be fierce, thus modulating demographic rates relative to the habitats in which coral densities are low. Together, these biotic effects could promote asynchronous dynamics of coral taxa among adjacent habitats in a cohesive reef-scape. Below we expand upon our treatment of the first hypothesized mechanism with the rationale that empirical evidence supports the existence of heterogeneous environmental conditions that are necessary for this mechanism to function.

### Association between coral community structure and the physical environment

The submarine relief of coral reefs juxtaposed with prevailing environmental conditions creates strong spatial heterogeneity in the effects of waves, currents, light, and seawater temperature^60^. Furthermore, for oceanic reefs like those surrounding Moorea, seawater circulation is driven by surface wave forcing, which creates spatiotemporal patterns in the residence time of seawater on the backreef^55^. The time that seawater spends in the shallow, biologically active backreef habitat will modulate physical (e.g., diurnal temperature range, DTR) and chemical (e.g., pH and O_2_ concentration) properties on the seawater flowing over the reef. Evidence for the spatial variability in environmental conditions at the present study site is seen in the discordance of DTR among habitats (pooled between depths on the fore reef), which might be a factor contributing to spatial variation in coral community structure.

The four habitats in which we have recorded asynchronous coral community dynamics are physically connected by wave-driven flow across the reef crest^55^. Surface gravity waves primarily break in shallow water (< 4 m) on the fore reef and at the reef crest, which results in a “piling” of seawater just behind the reef crest that forces a pressure-driven flow over the reef crest and into the lagoon^55^. This circulation pattern persists over a scale of ~ 1 km cross-reef, and it connects habitats through the transport of pelagic coral larvae^111^. It is reasonable to assume that increased wave flux increases the abundance of coral larvae in the back reef through delivery of larvae originating on the fore reef where coral populations typically are large, and can cover up to 81% (in 2019) of the benthos^52,70^. Therefore, periods of increased wave flux coincident with the availability of pelagic coral larvae (i.e., just after spawning) should favor elevated coral recruitment in the back reef at even fine spatial scales (< 1 km) where larval transport is facilitated by wave flux^91^. Evidence for this trend comes from the results of the simultaneous deployment of settlement tiles on the fore reef and in the back reef^112^, and the detection of coral larvae in seawater, including after it has passed over the reef crest^113,114^. Despite the current absence of empirical support for the coupling of wave forcing and coral recruitment in the back reef of Moorea, it is compelling to consider the possibility that occasional periods of wave-driven larval connectivity (e.g., 2005, 2012, 2014, and 2015) that lead to spatiotemporal asynchrony in coral community dynamics supports spatial insurance effects for these coral communities.

### Summary

Improving the understanding of the population dynamics of reef corals is an important goal in identifying the drivers of coral community resilience^44,53^. Exploring the mechanistic and causal drivers of spatiotemporal heterogeneity in scleractinian community structure (e.g., variable dispersal, environmental heterogeneity) is critical to understanding ecological resilience of coral reefs. As such, studies that examine the spatial grain of temporal variability in relation to the scale of biological (e.g., larval settlement) and environmental scales (e.g., factors that influence settlement and post-settlement processes), and the mechanisms that connect locations differing in these attributes, could provide an enhanced understanding of coral reef resilience. Progress towards this goal is likely to help explain the mechanistic basis of contrasting examples of coral community resilience, for example, between “oasis” and non-oasis sites^54^, or among different biogeographic regions^115^. While the resilience of coral communities is an emergent property of multiple processes^16,80^, the present study makes the case that spatial insurance represents one such process, the study of which, is likely to be beneficial in understanding coral community resilience. As contemporary time series that describe coral reef community structure extend to decadal scales, consideration of among-habitat insurance effects is becoming a tractable option to enrich the interpretation of archived data^33^. Such explorations can leverage the large investment of resources in coral reef monitoring to test hypotheses addressing causation for the ecological changes underway, and to more effectively design conservation projects with the best potential to ensure the persistence of coral reefs^5^.
